# *Nopalea cochenillifera* Regulates the Immune Response and Gut Microbiota in Mice

**DOI:** 10.3390/nu16244376

**Published:** 2024-12-19

**Authors:** Hana Kozai, Chihiro Watanabe, Rina Kosaka, Takafumi Aoki, Hiroki Hamada, Masato Kawashima, Takumi Kono, Kosuke Akagi, Michael J. Kremenik, Hiromi Yano, Mamoru Tanaka, Eri Oyanagi

**Affiliations:** 1 Graduate School of Bioscience and Biotechnology, Chubu University, Kasugai 487-8501, Japan; hkozai@isc.chubu.ac.jp (H.K.); gr22017-7137@sti.chubu.ac.jp (R.K.); m-tanaka@isc.chubu.ac.jp (M.T.); 2 Department of Health and Sports Science, Kawasaki University of Medical Welfare, Kurashiki 701-0193, Japan; chi.watanabe@mw.kawasaki-m.ac.jp (C.W.); h.hamada@mw.kawasaki-m.ac.jp (H.H.); masato.kawashima@mw.kawasaki-m.ac.jp (M.K.); wd224006@kwmw.jp (T.K.); wd224001@kwmw.jp (K.A.); yanohiro@mw.kawasaki-m.ac.jp (H.Y.); 3 Department of Clinical Nutrition, Kawasaki University of Medical Welfare, Kurashiki 701-0193, Japan; aoki.takafumi.54331@mw.kawasaki-m.ac.jp

**Keywords:** cactus, lipopolysaccharide, tumor necrosis factor-alpha, short-chain fatty acids, cellulose

## Abstract

Background: Cactus contains dietary fiber and minerals and is expected to have preventive effects against diabetes, arteriosclerosis, and other diseases. Additionally, cactus intake induces the production of short-chain fatty acids derived from the gut microbiota, which might influence immune functions. In this study, we examined the effects of a cactus (*Nopalea cochenillifera*: NC)-supplemented diet on lipopolysaccharide (LPS)-induced immune responses and intestinal barrier function. Methods: Male C3H/HeN mice were randomly divided into three groups—no fiber (NF), cellulose-containing fiber (Cellu), and cactus-added (NC) diets—for 6 weeks. The TNF-α and IL-10 responses to LPS, antibody titers, and intestinal barrier function, as well as the fecal microbiota, were analyzed. Results: The plasma TNF-α but not the IL-10 concentrations were significantly higher in the NC group than in the NF and Cellu groups. Furthermore, the plasma IgG antibody titers were significantly higher in the NC group than in the other groups. The NC group showed higher mucin content and IgA antibody titers in their feces compared with the Cellu group. The succinate and lactate contents, which induce a reduction in TNF-α secretion by macrophages, in the cecum of the NC group were significantly lower than those in the Cellu and NF groups. In contrast, the butyrate content was significantly higher in the cecum of the NC group compared to that of the Cellu group, with a significantly higher relative abundance of butyrate-producing bacteria. Conclusions: Taken together, we found that cactus intake regulates innate and adaptive immune function via the gut microbiota in mice. Therefore, cactus supplementation might serve as a strategy to develop novel functional foods with dietary fiber.

## 1. Introduction

In recent years, fears of food shortages due to global population growth [1] have demonstrated the need to develop and increase the production of a variety of food products [2]. Cactus has been described as a distinct plant that can be found in arid regions throughout the world and has been widely used in herbal medicines worldwide since ancient times [3]. The commercial production of cactus has advantages over other agricultural practices by mitigating damage to ecosystems and encouraging the adoption of sustainable agricultural practices [4]. Thus, it appears that cactus is considered to have immense potential to become a food of the future [5]. Many species of cactus have been used for a variety of purposes, such as for food, fodder, ornaments, and medicine. Cactus is nutritious and flavorful when eaten fresh: for example, its leaves can be used in vegetable and salad dishes, while its fruits can be made into juice [5]. Cactus contains dietary fiber and minerals and is expected to have preventive effects against diabetes, arteriosclerosis, and other diseases. The intake of cactus lowers low-density lipoprotein (LDL) cholesterol and prostaglandin [6], improves platelet function and hemostatic balance, and decreases the risk of atherosclerosis [7]. Additionally, cactus exhibits a rapid increase in high-density lipoprotein (HDL) cholesterol levels with a decrease in LDL cholesterol and triglycerides (TGs) in women affected by metabolic syndrome [8]. It also causes substantial reductions in body fat and total cholesterol in plasma and blood pressure and is beneficial for the body’s redox cardiovascular balance and type 2 diabetic conditions. However, information about the beneficial effects of cactus remains insufficient, as trials using cactus vary in terms of their methodology, design, and results [3].

*Nopalea cochenillifera*, which is a kind of cactus, is a species widely used in semiarid regions as a forage crop [9]. Although a previous study has reported the advantage of using *N. cochenillifera* in dietary supplements and functional foods because of the improvement in blood lipid parameters associated with cardiovascular risks [10], it is unclear whether *N. cochenillifera*, as a food high in dietary fiber, has a functional effect on immune function via the gut microbiota.

In recent years, the intestinal microbiome has gained attention as a symbiont influencing the host’s health, and the supply of dietary fiber is now considered an important factor in the formation of a healthy beneficial intestinal microbiome [11]. In fact, it has been reported that the active intake of dietary fiber induces the production of metabolites, such as short-chain fatty acids, via gut microbial fermentation [12], and can positively affect immune function [13,14]. Previously, we have reported that various low-molecular-weight dietary fibers (e.g., cellulose nanofiber [15,16] soybean residue: okara [17] and partially hydrolyzed guar gum: PHGG [12,14]) have positive effects on the intestinal microbiota including changes in the relative abundance of the bacterial community, increases in short-chain fatty acid (SCFA) production by the gut microbiota [12], and also attenuation of proinflammatory cytokine production [14].

In this study, by using a processed powdered form of *N. cochenillifera* as a cactus food, we examined the effects of a cactus-added diet on the immune and intestinal barrier function in mice. Our study presents a strategy to expand the use of *N. cochenillifera* that might be developed as one a source of dietary fiber, especially as an immune-regulatory food for health.

## 2. Materials and Methods

### 2.1. Animals and Experimental Conditions

Nine-week-old male C3H/HeN mice (*n* = 34) were purchased from CLEA Japan (Tokyo, Japan). Three to four mice were housed in a cage and maintained on a 12:12 h light/dark cycle with food and water available ad libitum. The animals were group housed to minimize stress confounders. The mouse experiment, including all conducted procedures, was approved by the Institutional Animal Care and Use Committee of Kawasaki University of Medical Welfare (22-004).

### 2.2. Food and Drugs

The mice were fed a non-fiber (NF) control diet (D12450Jpx1, Research Diets, New Brunswick, NJ, USA) containing 10% fat, 20% protein, and 70% carbohydrates (of total calories). The diet also contained two conditions: 5% cellulose (Cellu) diet (D12450J, Research Diets, New Brunswick, NJ, USA) and 5% powder *N. cochenillifera* diet (NC). *N. cochenillifera* cladodes were grown in a greenhouse at Chubu University in Kasugai, Aichi Prefecture, Japan. Young cladodes were harvested from plants aged 3 years. The harvested cladodes were cut into smaller pieces, freeze-dried, and milled. Then the powder added with the non-fiber diet and mixed well using a stand mixer (WSM7Q, Waring, Stamford, CT, USA) for use in the following experiments (Table S1). LPS (*Escherichia coli* 055: B5) was purchased from Sigma-Aldrich (St. Louis, MO, USA).

### 2.3. Plasma Cytokines in Response to LPS

The mice were randomly divided into three groups: NF (*n* = 10), Cellu (*n* = 12), and NC (*n* = 12) groups. Sample sizes were determined based on the existing literature of similar studies [14,18]. As an indicator of the intestinal environment, the fecal pH was measured at 6 weeks, and IgA antibody titers in the feces were measured from collected stool for 3 days in all mice. After each 6-week dietary intervention, the mice were lightly anesthetized with isoflurane, followed by an injection of LPS (1 mg/kg) into the orbital vein [19]. Blood samples were collected at 1 h after LPS injection to measure the plasma concentration of TNF-α as a proinflammatory cytokine and interleukin (IL)-10 as an anti-inflammatory cytokine. After blood collection, the colons were collected to evaluate the gene expression of tight junctions and immunological parameters (Table S2).

### 2.4. Enzyme-Linked Immunosorbent Assay for Cytokines

Plasma TNF-α and IL-10 concentrations were measured via enzyme-linked immunosorbent assay (ELISA) using commercially available kits (Mouse TNF-α and IL-10 Quantikine ELISA Kit, R&D Systems, Minneapolis, MN, USA).

### 2.5. Real-Time Quantitative PCR

Total RNA was extracted using TRIzol Reagent (Invitrogen, Carlsbad, CA, USA) and RNeasy Mini Kit (Qiagen, Valencia, CA, USA). RNA purity was assessed using the NanoDrop system (Nano Q5 Technologies, Wilmington, DE, USA). RT-PCR was performed using the reverse transcription Kit (Applied Biosystems, Waltham, MA, USA) and the step one plus real-time PCR system (Applied Biosystems) with Fast SYBR Green PCR master Mix Kits (Applied Biosystems). The following amplification procedure was applied: initial denaturation for 10 min at 95 °C, followed by 40 cycles of denaturation for 3 s at 95 °C and annealing for 15 s at 60 °C. As the housekeeping gene, we used glyceraldehyde-3-phosphate dehydrogenase (*Gapdh*) mRNA, and all data are represented relative to its expression, using standard curve methods. The specific PCR primer pair for the studied genes are shown in Table S2.

### 2.6. Fecal Mucin Content and IgA Titer Assays

For fecal volume, fecal samples were collected for 24 h over a 3-day period and stored at −20 °C for a period of time. The feces were then freeze-dried and milled for the measurement of fecal weight and mucin in the feces. Fecal mucin contents were determined using a fluorometric assay kit (Fecal Mucin Assay Kit: FFA-MU-K01, Cosmobio, Tokyo, Japan), which discriminates O-linked glycoproteins (mucins) from N-linked glycoproteins. The immunoglobulin (Ig) A in feces and the IgA, IgG, and IgM in serum were determined via ELISA using IgA, IgG, and IgM ELISA kits for their respective quantitative analyses (Invitrogen).

### 2.7. Gut Microbiota

Collected fresh fecal samples from the mouse colon were immediately frozen in liquid nitrogen and stored at −80 °C. Bacterial DNA was extracted from the frozen fecal samples using a QIAamp Fast Stool DNA Mini Kit (Qiagen). The extracted bacterial DNA was subjected to amplicon sequence analysis using the MiSeq system (Illumina, San Diego, CA, USA) by Techno Suruga Lab. DNA was extracted using an automated DNA isolation system (GENEPREPSTARPI-480, Kurabo, Osaka, Japan), with 200 μL of distilled water included as a negative control sample.

The V3–V4 regions of Prokaryote 16S rRNA were amplified from the extracted DNA using the Pro341F/Pro805R primers and the dual index method. A negative control sample was also included, and the amplicons were visualized by electrophoresis.

Pro341F: 5′-CCTACGGGNBGCASCAG-3′

Pro805R: 5′-GACTACNVGGGTATCTAATCC-3′

Barcoded amplicons were paired-end sequenced on a 2 × 284 bp cycle using the MiSeq system with MiSeq Reagent Kit v3 (15 Gb: 600 Cycle). The quality of the paired-end sequencing reads was checked using the FASTX-Toolkit, v0.0.14, and they were merged using the fastq-join program with the default settings. The joined reads extracted a quality value score (QC) of ≥20 for more than 99% of the sequence. The sequences’ homology of ≥97% identity were clustered into the same bacterial species identification using the Metagenome@KIN Ver 2.2.1 analysis software (World Fusion, Tokyo, Japan) and the TechnoSuruga Lab Microbial Identification database DB-BA 13.0 (TechnoSuruga Laboratory, Shizuoka, Japan). The 16S rRNA data were analyzed with Quantitative Insights into Microbial Ecology (QIIME) 2.0 ver. 2022.6 [20]. Quality filtering and chimeric sequences were filtered using DADA2 (Divisive Amplicon Denoising Algorithm 2) denoise-single plugin ver. 2017.6.0 with the default option [21]. The taxonomy was assigned using the Silva database ver. 138 based on an average percent identity of 99% [22]. For analyzing β-diversity, unweighted UniFrac distance metrics were used. We carried out principal coordinates analysis (PCoA) to show the pattern of differences. The α-diversity was calculated using the amplicon sequence variants (ASVs), Chao 1 [23], Shannon [24], and Simpson [25] indices.

### 2.8. Succinate, Lactate, and SCFAs in the Cecum

The concentration of organic acids in the cecum contents was determined via a high-performance liquid chromatographic method using a Prominence CDD-10Avp conductivity detector (Shimadzu Corporation, Kyoto, Japan) with a post-column reaction in tandem columns [26].

### 2.9. Statistical Analysis

The data of cytokines, mRNA expressions levels, mucin, IgA and IgG titers, organic acids, and microbial α-diversities are expressed as bee swarm and boxplots. We performed statistical calculations (data visualization included) using R ver. 4.3.1. The data were analyzed using one-way analysis of variance (ANOVA) or the Kruskal–Wallis test after the Shapiro–Wilk test. Post hoc tests were conducted as follows: the Bonferroni test was carried out after the ANOVA test, and the Steel–Dwass test was carried out after the Kruskal–Wallis test. We analyzed the statistical distances among the three groups in β-diversity using permutational multivariate analysis of variance (PERMANOVA). PERMANOVA was performed in R Studio (ver. 2023.03.02) with the vegan package using the ASV-level Unifrac distance. For each case, a *p* value < 0.05 was considered statistically significant. A volcano plot (cut log2 fold-change = 5 and cut *p* = 0.05) was applied for visualizing the expression of the differentially expressed microbiota (species levels) for screening.

## 3. Results

### 3.1. Effect of NC Intake on Body Weight, Cytokine Responses to LPS, and Immunoglobulin Titers

**Figure 1:**
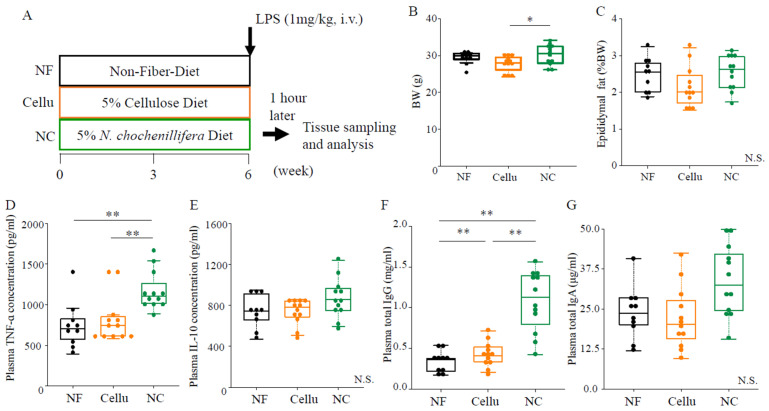
The experimental design and the effects of NC intake on body composition and plasma immunological parameters: Experimental protocol (**A**), body weight (**B**), epididymal fat (**C**), plasma TNF-*α* (**D**), and IL-10 (**E**) concentrations in response to LPS administration and plasma IgG (**F**) and IgA (**G**) titers. *: *p* < 0.05, **: *p* < 0.01 and N.S.: not significant, determined to conform normality using the Shapiro-wilk test, and to perform the one-way ANOVA with Bonferroni’s post hoc test (**C**–**E**) or the Kruskal-Wallis test with Streel-Dwass post hoc test (**B**,**F**,**G**).

In this experiment (Figure 1A), the body weight in the NC mice was slightly but significantly higher than that in the Cellu mice (*p* < 0.05, Figure 1B), although no excess body fat accumulation was observed (Figure 1C). The plasma TNF-α concentration in the NC group after LPS administration was significantly higher than that in both the NF (*p* < 0.01) and Cellu (*p* < 0.01) mice (Figure 1D). On the other hand, the plasma IL-10 concentration was not different among the three groups (Figure 1E). The plasma IgG titer in the Cellu group was significantly higher than that in the NF group (*p* < 0.01). In addition, the IgG titer in the NC group was significantly higher than that in both the NF (*p* < 0.01) and Cellu (*p* < 0.01) groups (Figure 1F). The plasma IgA level was not different among the three groups (Figure 1G).

### 3.2. Effect of NC Intake on Intestinal Environment

**Figure 2:**
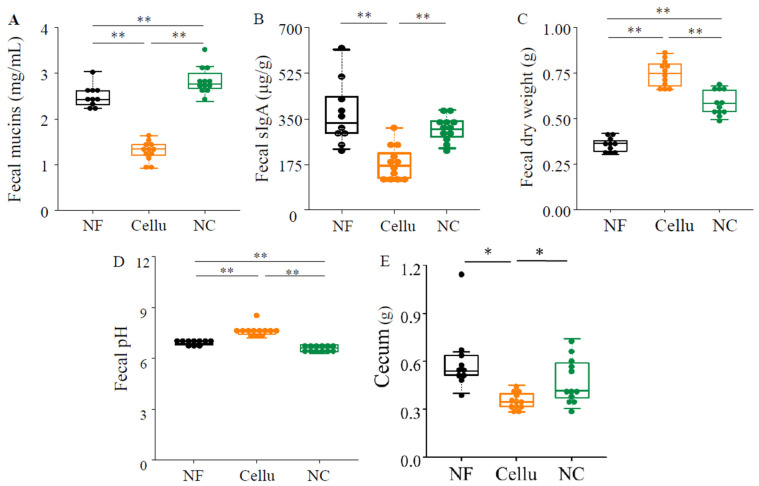
The effect of NC intake on intestinal barrier factors’ mucins content (**A**), sIgA (**B**), fecal dry weight (**C**), pH (**D**), and cecum content (**E**) in feces *: *p* < 0.05 and **: *p* < 0.01, determined to conform normality using the Shapiro-wilk test, and to perform the one-way ANOVA with Bonferroni’s post hoc test (**B**,**C**) or the Kruskal-Wallis test with Streel-Dwass post hoc test (**A**,**D**,**E**).

No effect of NC intake on the mRNA expression of tight junctions involved in the permeability of intestinal epithelial cells was observed (Figure S1A–H). Mucin, the main component of mucus secreted by epithelial cells, which is involved in the protective action of the intestinal tract, was, however, highest in the NC group and lowest in Cellu group among the three groups (Figure 2A). Secretory IgA, which is secreted from the surface of the intestinal mucosa into the intestinal lumen, was significantly higher in the NC and NF groups, compared to the Cellu group (*p* < 0.01, Figure 2B). In contrast, the fecal dry weight was highest in the Cellu group, followed by the NC and NF groups (*p* < 0.01, Figure 2C).

The fecal pH in the NC group was the lowest of the three groups (Figure 2D), suggesting a high product of organic acids by gut microbiota. Moreover, the cecum content in NF and NC mice was higher than in the Cellu group (*p* < 0.05, respectively, Figure 2E), suggesting changes in the gut microbial diversity.

### 3.3. Effect of NC Intake on Organic Acid Contents and Gut Microbiota

**Figure 3:**
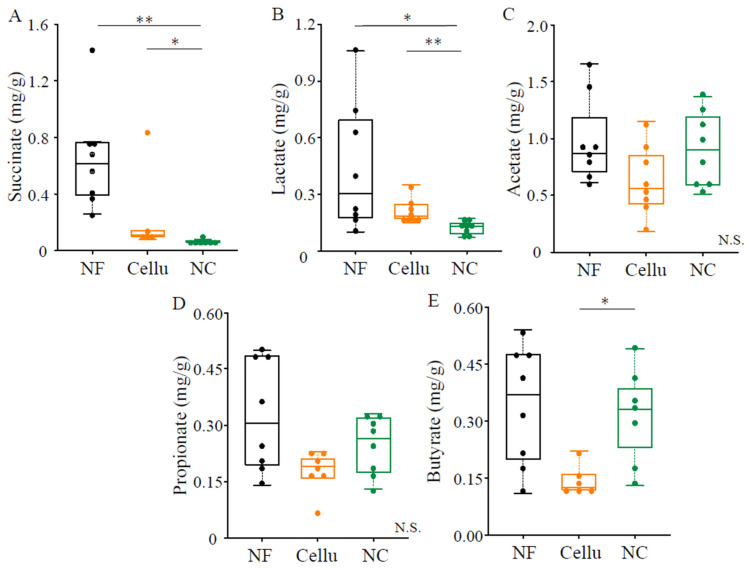
The effects of NC intake on the contents of succinate (**A**), lactate (**B**), and SCFAs (acetate (**C**), propionate (**D**), and butyrate (**E**)) in the cecum of mice. *: *p* < 0.05, **: *p* < 0.01 and N.S.: not significant, determined to conform normality using the Shapiro-wilk test, and to perform the one-way ANOVA with Bonferroni’s post hoc test (**C**) or the Kruskal-Wallis test with Streel-Dwass post hoc test (**A**,**B**,**D**,**E**).

Indeed, succinic acid (Figure 3A), lactate (Figure 3B), and short-chain fatty acids, including acetate (Figure 3C), propionate (Figure 3D), and butyrate (Figure 3E), were detected as organic acids in cecum. In particular, the lowest levels of succinic and lactate among the three groups were observed in NC mice (Figure 3A,B). On the other hand, a content of butyrate, which has been reported to have anti-inflammatory and immune cell effects, was significantly higher in the NC group compared to the Cellu group (*p* < 0.05, Figure 3E).

**Figure 4:**
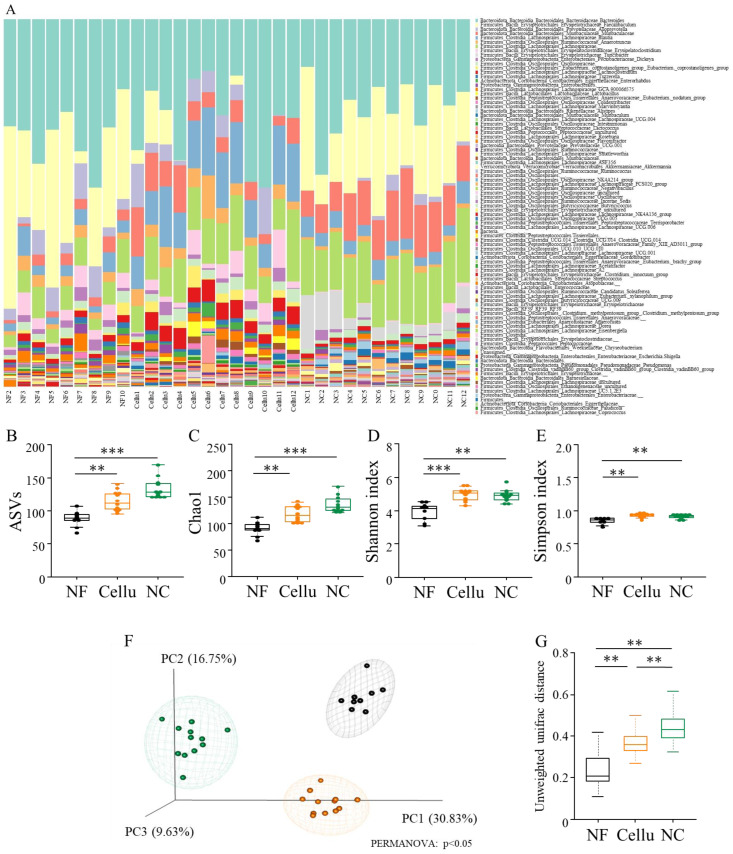
Gut microbial comparisons among NF, Cellu, and NC mice and the effect of NC intake on microbiota characterization. Relative abundance of the gut microbiota determined using 16S rRNA sequences in NF, Cellu, and NC at the genus level (**A**). The α-diversity indices (including ASVs (**B**), Chao1 (**C**), Shannon (**D**), and Simpson (**E**) indices) and β-diversity indices (**F**) (including UniFrac distance (**G**)) are shown. **: *p* < 0.01 and ***: *p* < 0.001. Differences in α-diversity indices were analyzed using the Streel-Dwass test following the Kruskal-Wallis test.

**Figure 5:**
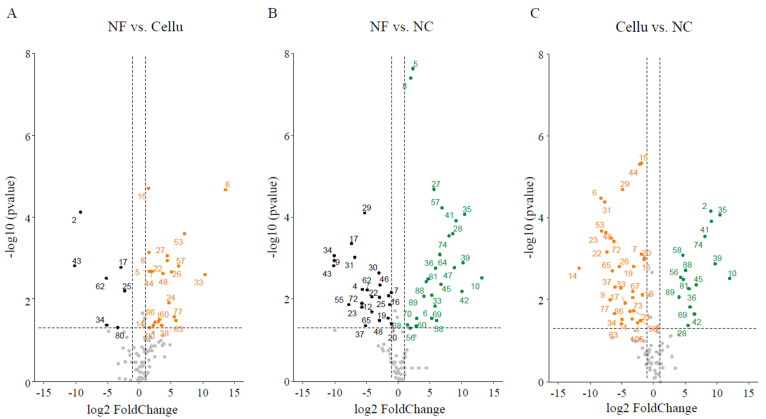
The comparison among gut microbiota in NF, Cellu, and NC mice using volcano plots. The *p* values were plotted against dietary fiber–induced changes in gut microbiota of mice collected from NF vs. Cellu (**A**), NF vs. NC (**B**), and Cellu vs. NC (**C**) groups. Black, orange, and green dots show that the bacterial abundances were significantly elevated/depressed (*p* < 0.05) for each group, respectively, and dots in gray indicate unchanged bacterial abundances.

Furthermore, we evaluated the diversities to assess the impact of the NC on the gut microbiota (Figure 4). The gut microbiota α-diversities in the NC and Cellu groups were observed to be significantly higher than those in the NF group: ASVs (*p* < 0.01, Figure 4B), Chao1 (*p* < 0.01, Figure 4C), Shannon (*p* < 0.01, Figure 4D), and Simpson (*p* < 0.01, Figure 4E). The α-diversities, however, was not significantly difference between in the NC and Cellu groups (Figure 4B–E). As a result of the β-diversity evaluation, three clusters were formed with or without fiber diets (NF vs. Cellu and NC), and with different types of fiber (Cellu vs. NF) intakes (PERMANOVA: *p* < 0.05, Figure 4F), where each group was significantly different from the other groups based on the UniFrac distances (*p* < 0.01, respectively, Figure 4G). The stacked bar plots in Figure 4A indicate the proportion of the relative abundances of gut microbiota at the genus level in each sample. Through visualization using volcano plots, high abundance levels of gut microbes in the NC group compared with the NF and Cellu groups were extracted for 16 species: *s_Faecalibaculum rodentium*, *g_Turicibacter*, *s_Alistipes_finegoldii*, *g_Prevotellaceae UCG-001*, *g_Alistipes*, *g_Shuttleworthia*, *g_ASF356*, *g_Roseburia*, *s_Ruminococcus champanellensis*, *g__Lachnospiraceae UCG-006*, *g_Incertae_Sedis*, *g_Lachnospiraceae_UCG-001*, *g__A2*, *s_Trichinella pseudospiralis*, *s__[Clostridium] leptum*, and *g_[Eubacterium]_xylanophilum_group* (2, 10, 28, 35, 36, 39, 41, 42, 45, 56, 58, 69, 74, 81, 88, and 89, respectively, Figure 5).

## 4. Discussion

The effects of NC intake, as part of a powdered cactus-added diet, on immune function and intestinal barrier function in mice were investigated. Not only were innate and adaptive immune functions enhanced but also the intestinal barrier function. Additionally, with NC intake, butyrate production was increased via changes in the gut microbiota, whereas succinate and lactate were regulated at low levels.

It is well known that LPS promotes nuclear translocation of the transcription factor NF-κB via Toll-like receptors 4 (TLR4), which represent the innate immune system, as well as inducing the expression of inflammatory cytokine genes [27]. In this study, TNF-α production in response to LPS was increased by *N. cochenillifera* intake. In addition, no significant changes were observed in the anti-inflammatory cytokine IL-10, indicating that *N. cochenillifera* intake may shift the innate immune function to a higher innate immune response, at least with respect to bacterial infection. As the present study was not conducted in mouse models of metabolic diseases such as obesity and diabetes, the main TNF-α-producing cells appeared to be immune cells, such as macrophages [19], other than inflammatory immune cells in adipose tissue [28,29]. Therefore, it is suggested that *N. cochenillifera* intake may cause an increase in bio-protection against infection by bacteria (e.g., Gram-negative bacteria). In vitro studies on various cactus extracts have reported several functions such as anti-microbial potential, antioxidant capacity, and anti-biofilm activity [30,31,32]. To the best of our knowledge, however, there have been no reports on *N. cochenillifera*. In vivo studies have investigated the anti-tumor effects of cactus extract on lymphoma [33] and anti-inflammatory effects on Euphorbiaceae-induced inflammation (rash) [34]. Although it is clear that *N. cochenillifera* contains soluble fiber, even though it has a high molecular weight (Table S1), we have yet to identify the dietary fiber that affects the innate immune system via fermentation products formed by the gut microbiota. Thus, further research is warranted.

**Figure 6:**
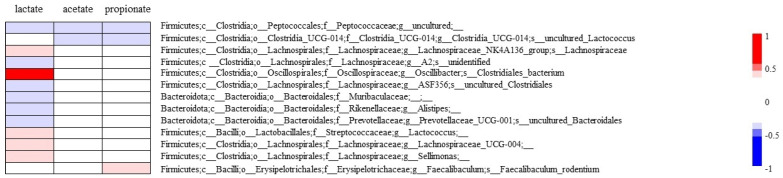
The heatmap derived from the correlation matrix between organic acids (lactate, acetate, and propionate) and the relative abundance of only the genera and species of bacteria that were significantly correlated with any of the three organic acids are shown. The darker red bar indicates a strongly positive correlation (towards r = 1.0), while the darker blue bar indicates a strongly negative correlation (towards r = −1.0). The represented white regions are not significant at *p* ≥ 0.05.

Succinate acts in synergy with TLR ligands to produce proinflammatory cytokines through GPR91, as well as enhancing the antigen-specific activation of human and mouse helper T cells [35]. Recent studies have, however, indicated that succinate can suppress immune responses; for example, it suppressed the secretion of inflammatory mediators IL-6, TNF-α and nitric oxide (NO), as well as inhibited *Il1β* and *iNos* mRNA expressions of inflammatory macrophages in a GPR91-independent manner [36]. Furthermore, it has been shown that lactate also suppresses the macrophage proinflammatory response to LPS stimulation via GPR81-mediated signaling [37,38,39]. In this line, our results indicated that the suppression of succinate and lactate after *N. cochenillifera* intake was associated with secretion of TNF-α of macrophages in response to LPS. Additionally, a recent study reported a positive correlation between *Alistipes* genus and TNF-α production [40]. TNF upregulation was due to the role of proinflammatory Gram-negative bacteria, *Alistipes*, binding to TLR4, priming the expression of TNF production [41]. In fact, we observed negative correlations between *Alistipes* abundances and lactate contents (Figure 6). Thus, TNF-α production in response to LPS led to reductions in two factors—succinate and lactate—and an increase in one bacterium—*Alistipes*—after *N. cochenillifera* intake. These alterations, as a synergistic effect on the innate immune function, might be induced by *N. cochenillifera* intake.

In addition, the effects of *N. cochenillifera* intake on adaptive immunity were examined; in particular, in terms of IgG antibody production, which is important for the defense function of the body. Although antibody titers to specific antigens were not evaluated in the present study, total IgG antibody titers in plasma were found to be at least elevated with *N. cochenillifera* intake. An increase in the specific immune function (serum IgM and IgG levels) in mice after intraperitoneal administration of a polysaccharide extracted from the cactus *Opuntia dilleniid* has been reported [42]. This is, however, the first report of an increase in antibody titer after *N. cochenillifera* intake. Moreover, our results also suggested that *N. cochenillifera* intake enhanced the intestinal barrier function. We observed that the levels of mucin and sIgA in the feces were clearly different from those associated with a normal diet containing cellulose. Mucin, which forms the intestinal mucosal layer, is a secretion, which plays the role of a physical barrier for the intestinal tract (acting as the front line of host defense against bacteria and foreign antigens) and a useful habitat for resident bacteria [43]. Conversely, the composition of the gut microbiota is also an important factor contributing to the regulation of intestinal mucus barrier function. As the gut microbial community plays an important role in influencing intestinal mucus [44], we expected to observe interesting results related to changes in the gut microbiota. Indeed, *N. cochenillifera* induced an increase in the relative abundance of butyrate-producing bacteria, leading to an increase in the content of butyrate, as well as changes in the α- and β-diversities of gut microbiota. Dietary fiber is fermented by the gut microbiota to organic acids such as SCFA, some of which are absorbed by the intestinal cells. The organic acids are then distributed via the portal vein to peripheral tissues such as skeletal muscle, liver, adipose tissue, and the brain, where they bind to specific receptors (e.g., GPR41, GPR43, GPR109a, GPR81, GPR91, etc.) to exert their effects [45,46]. Thus, it is possible that dietary fiber in *N. cochenillifera* is fermented to organic acids containing SCFAs by the gut microbiota.

In this study, *N. cochenillifera* intake-induced butyrate-producing bacteria included *Shuttleworthia*, *Clostridium* sp. *ASF356*, *Roseburia*, and *Clostridium leptum*. Hence, it was suggested that butyrate from these bacteria may have influenced the immune system. It is known that SCFAs modulate the immune system through the stimulation of various immune cells [47]. Indeed, SCFAs induce the migration of neutrophil to inflammatory sites and then promote phagocytosis [48]. Furthermore, SCFAs regulate T cell differentiation (Th1, Th17, and Treg) [48]. In addition, SCFAs modulate intestinal barrier function by inducing the secretion of IL-18, anti-microbial peptides, and mucins from intestinal epithelial cells and increasing the expression of tight junction molecules [47]. It has also been suggested that SCFAs may induce intestinal IgA production [47], thus leading to an enhancement effect of sIgA production, which is consistent with our results. To prevent the translocation of commensal bacteria and enteric pathogens across the large intestine epithelium, it appears that the host has developed multiple defense mechanisms including the mucin, which is a highly glycosylated protein [49], and sIgA, which plays a major role in protecting mucosal surfaces against colonization and possible invasion by pathogenic microorganisms [50]. Gamage et al. (2020) suggested that specific associations between gut microbes and mucin structures may be identified [49]. Additionally, it is clear that sIgA plays a crucial role in shaping host–microbiota interactions [51]. Although we were not able to confirm the induction of T cell differentiation, which is a limitation of the study, further investigation should elucidate the mechanism of immune induction after *N. cochenillifera* intake. At the least, our results suggest that *N. cochenillifera* as a new functional food with cactus components, might act as an intestinal barrier and play a role in immune functions.

## 5. Conclusions

We found that the powdered *N. cochenillifera*-added diet regulates innate and adaptive immune functions and intestinal barrier function. These phenomena might be caused by a dramatic suppression of succinate and lactate productions via changes in the gut microbiota.

## Data Availability

The data that support the findings of this study are not openly available due to reasons of sensitivity and are available from the corresponding author upon reasonable request.

## Supplementary Materials

The following supporting information can be downloaded at: https://www.mdpi.com/article/10.3390/nu16244376/s1, Figure S1. The mRNA expression of the intestinal tight junctions Zo-1 (A), Cldn4 (B), Occludin (C), Cldin5 (D), Cldin3 (E), Cldin7 (F), Cldin1 (G), and Muc2 (H); Figure S2. The mRNA expression of immune cell markers in the large intestine Tnf-α (A), F4/80 (B), Cd11c (C) and Cd163 (D). **: *p* < 0.01; Table S1. Composition of *N. cochenillifera*; Table S2. Primer sequences for Real-time RT-PCR analysis; Table S3. Code list of the volcano plots (Figure 5) of the gut microbiota.
