# Supplementary material for: Nopalea cochenillifera Regulates the Immune Response and Gut Microbiota in Mice
Source: Nutrients. 2024 Dec 19;16(24):4376. doi: 10.3390/nu16244376 (PMC11677944; doi:10.3390/nu16244376)
Supplement: Supplementary file 1 [file nutrients-16-04376-s001.zip › nutrients-3301089-supplementary.pdf]

Supplemental data

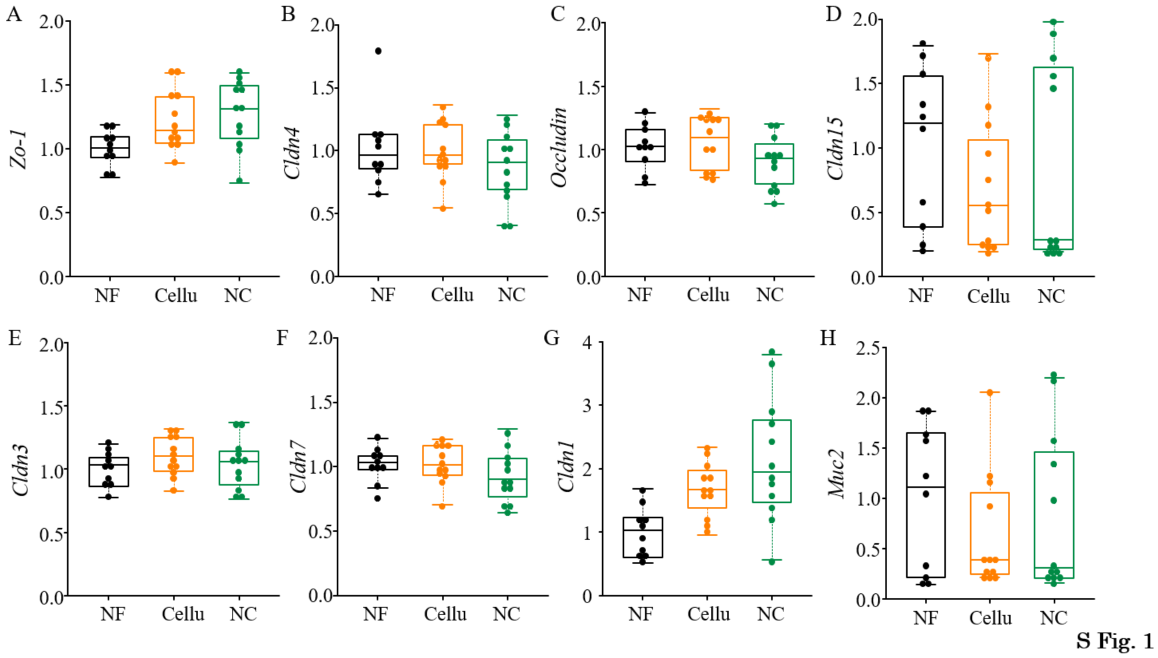

Figure S1. The mRNA expression of the intestinal tight junctions Zo-1 (A), Cldn4 (B), Occludin (C), Cldin5 (D), Cldin3 (E), Cldin7 (F), Cldin1 (G), and Muc2 (H).

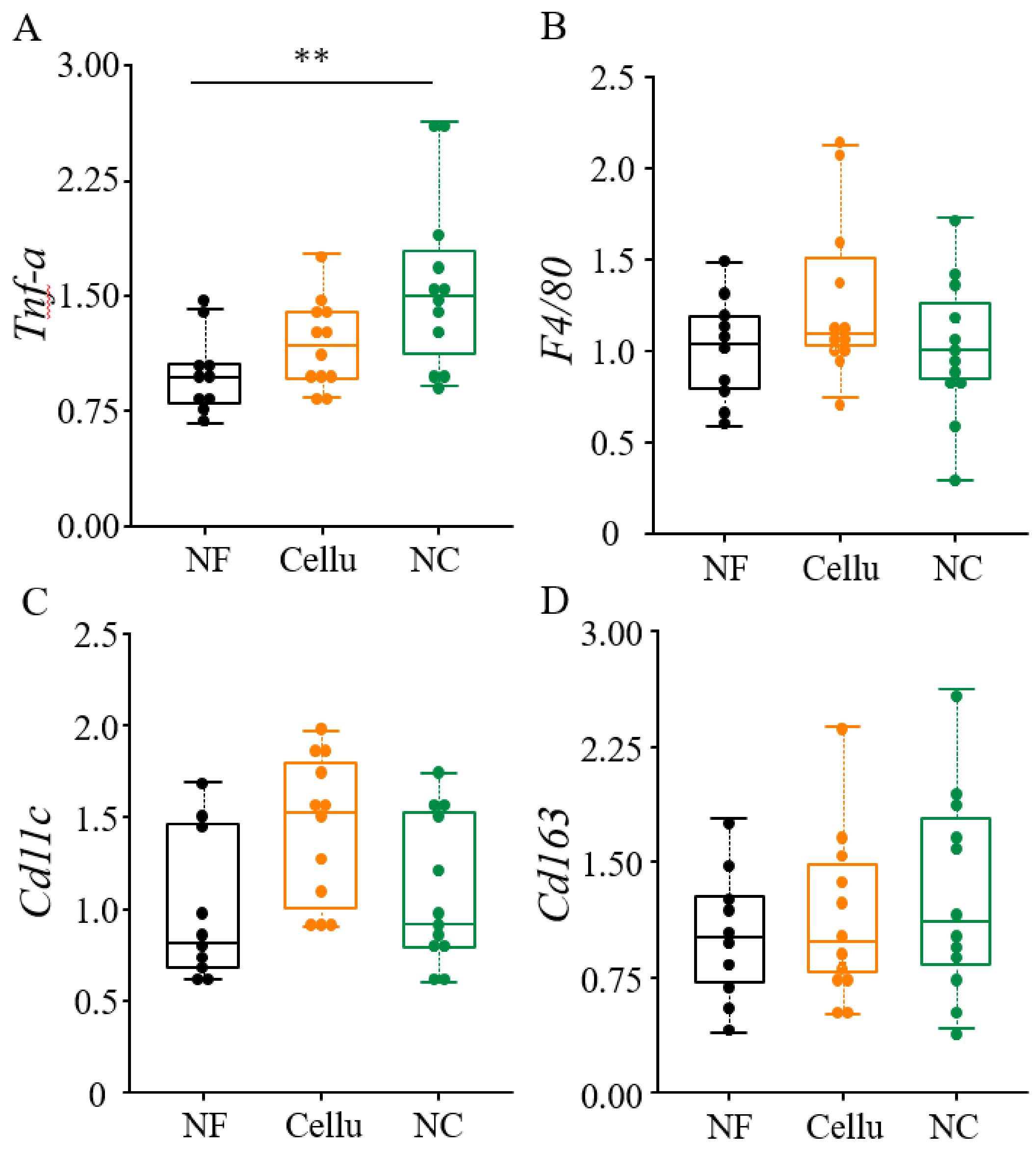

Figure S2. The mRNA expression of immune cell markers in the large intestine Tnf-*α* (A), F4/80 (B), Cd11c (C) and Cd163 (D). **: $p < 0.01$.

Table S1. Composition of *N. cochenillifera*.

Parameter	Contents in powder *N. cochenillifera*
Water (mg/g)	38
Protein (mg/g)	119
Lipid (mg/g)	29
Mineral (mg/g)	163
Carbohydrate (mg/g)	651
Suger (mg/g)	174
Dietary fiber (mg/g)	477
Insoluble dietary fiber (mg/g)	378
High molecular weight soluble dietary fiber (mg/g)	99
Low molecular weight soluble dietary fiber (mg/g)	<5
Energy (kcal/g)	2.4
Sodium (μg/g)	45
Sodium chloride equivalent (μg/g)	110

Table S2. Primer sequences for Real-time RT-PCR analysis.

Gene	Forward	Reverse
Zo-1	CGCGGAGAGAGAGACAAGATGT	TCCTCCATTGCTGTGCTCTT
Occludin	TCCGTGAGGCCTTTTGAA	GGTGCATAATGATTGGGTTTG
Claudin1	CCACCATTGGCATGAAGTGC	GGCCAAATTCATACCTGGCA
Claudin3	GCAAGCAGACTGTGTGTCGT	TACCGTCACCACTACCAGCA
Claudin4	GGCGTCTATGGGACTACAGG	GAGCGCACAACTCAGGATG
Claudin7	GCTAAGAAGCCCAACACCAG	TGCAAAATGTACGACTCGGT
Claudin15	GCTTCTTCATGTCAGCCCTG	TTCTTGGAGAGATCCATGTTGC
Mutin2	ACAAGCTGGCAGTGGTGAA	TGTCCAGCTCCACCATGAG
F4/80	CTTTGGCTATGGGCTTCCAGTC	GCAAGGAGGACAGAGTTTATCGTG
CD11c	CTGGATAGCCTTTCTTCTGCTG	GCACACTGTGTCCGAACTC
CD163	GGGTCATTCAGAGGCACACTG	CTGGCTGTCCTGTCAAGGCT
Tnf-α	ACTTGGTGGTTTGCTACGAC	CCTCCCTCTCATCAGTTCTA
Il-1β	AAAAAAGCCTCGTGCTGTCG	GTCGTTGCTTGGTTCTCCTTG
Gapdh	TGAAGCAGGCATCTGAGGG	CGAAGGTGGAAGAGTGGGAG

Table S3. Code list of the volcano plots (Fig. 5) of the gut microbiota.

Code	Phylum	Class	Order	Family	Genus	Species
2	p__Firmicutes	c__Bacilli	o__Erysipelotrichales	f__Erysipelotrichaceae	g__Faecalibaculum	s__Faecalibaculum_rodentium
4	p__Bacteroidota	c__Bacteroidia	o__Bacteroidales	f__Prevotellaceae	g__Alloprevotella	s__uncultured_Bacteroidales
5	p__Bacteroidota	c__Bacteroidia	o__Bacteroidales	f__Muribaculaceae	g__Muribaculaceae	s__uncultured_bacterium
6	p__Firmicutes	c__Clostridia	o__Lachnospirales	f__Lachnospiraceae	g__Blautia	s__uncultured_Clostridiales
7	p__Firmicutes	c__Clostridia	o__Oscillospirales	f__Ruminococcaceae	g__Anaerotruncus	s__Anaerotruncus_sp.
8	p__Firmicutes	c__Clostridia	o__Lachnospirales	f__Lachnospiraceae	__	__
9	p__Firmicutes	c__Bacilli	o__Erysipelotrichales	f__Erysipelatoclostridiaceae	g__Erysipelatoclostridium	__
10	p__Firmicutes	c__Bacilli	o__Erysipelotrichales	f__Erysipelotrichaceae	g__Turicibacter	__
12	p__Proteobacteria	c__Gammaproteobacteria	o__Enterobacterales	__	__	__
14	p__Firmicutes	c__Clostridia	o__Oscillospirales	f__[Eubacterium]_coprostanoligenes_group	g__[Eubacterium]_coprostanoligenes_group	__
15	p__Firmicutes	c__Clostridia	o__Lachnospirales	f__Lachnospiraceae	g__Lachnoclostridium	__
16	p__Firmicutes	c__Clostridia	o__Lachnospirales	f__Lachnospiraceae	g__Tuzzerella	s__uncultured_organism
17	p__Firmicutes	c__Clostridia	o__Lachnospirales	f__Lachnospiraceae	g__Sellimonas	__
18	p__Actinobacteriota	c__Coriobacteriia	o__Coriobacteriales	f__Eggerthellaceae	g__Enterorhabdus	s__Adlercreutzia_muris
19	p__Firmicutes	c__Bacilli	o__Erysipelotrichales	f__Erysipelatoclostridiaceae	g__Erysipelatoclostridium	s__unidentified
20	p__Firmicutes	c__Clostridia	o__Lachnospirales	f__Lachnospiraceae	g__GCA-900066575	s__uncultured_Clostridium
21	p__Firmicutes	c__Bacilli	o__Lactobacillales	f__Lactobacillaceae	g__Lactobacillus	__
22	p__Firmicutes	c__Clostridia	o__Peptostreptococcales-Tissierellales	f__Anaerovoracaceae	g__[Eubacterium]_nodatum_group	__
23	p__Firmicutes	c__Clostridia	o__Lachnospirales	f__Lachnospiraceae	g__Blautia	__
24	p__Firmicutes	c__Clostridia	o__Oscillospirales	f__Oscillospiraceae	g__Colidextribacter	s__Firmicutes_bacterium
25	p__Firmicutes	c__Clostridia	o__Lachnospirales	f__Lachnospiraceae	g__Marvinbryantia	s__Clostridiales_bacterium
26	p__Firmicutes	c__Clostridia	o__Lachnospirales	f__Lachnospiraceae	g__Lachnoclostridium	s__Clostridium_fusiformis
27	p__Bacteroidota	c__Bacteroidia	o__Bacteroidales	f__Muribaculaceae	g__Muribaculum	s__Muribaculum_intestinale
28	p__Bacteroidota	c__Bacteroidia	o__Bacteroidales	f__Rikenellaceae	g__Alistipes	s__Alistipes_finegoldii
29	p__Firmicutes	c__Clostridia	o__Lachnospirales	f__Lachnospiraceae	g__Lachnospiraceae_UCG-004	__
30	p__Firmicutes	c__Bacilli	o__Lactobacillales	f__Streptococcaceae	g__Lactococcus	__
31	p__Firmicutes	c__Clostridia	o__Oscillospirales	f__Oscillospiraceae	g__Intestinimonas	__
33	p__Firmicutes	c__Clostridia	o__Lachnospirales	f__Lachnospiraceae	g__Roseburia	s__Eubacterium_plexicaudatum
34	p__Firmicutes	c__Clostridia	o__Oscillospirales	f__Oscillospiraceae	g__Flavonifractor	__
35	p__Bacteroidota	c__Bacteroidia	o__Bacteroidales	f__Prevotellaceae	g__Prevotellaceae_UCG-001	s__uncultured_Bacteroidales
36	p__Bacteroidota	c__Bacteroidia	o__Bacteroidales	f__Rikenellaceae	g__Alistipes	__
37	p__Firmicutes	c__Clostridia	o__Oscillospirales	f__Oscillospiraceae	g__Colidextribacter	__
38	__	__	__	__	__	__
39	p__Firmicutes	c__Clostridia	o__Lachnospirales	f__Lachnospiraceae	g__Shuttleworthia	s__uncultured_Roseburia

40	p__Bacteroidota	c__Bacteroidia	o__Bacteroidales	f__Muribaculaceae	__	__
41	p__Firmicutes	c__Clostridia	o__Lachnospirales	f__Lachnospiraceae	g__ASF356	s__uncultured_Clostridiales
42	p__Firmicutes	c__Clostridia	o__Lachnospirales	f__Lachnospiraceae	g__Roseburia	__
43	p__Verrucomicrobiota	c__Verrucomicrobiae	o__Verrucomicrobiales	f__Akkermansiaceae	g__Akkermansia	s__Akkermansia_muciniphila
44	p__Firmicutes	c__Clostridia	o__Oscillospirales	f__Ruminococcaceae	__	__
45	p__Firmicutes	c__Clostridia	o__Oscillospirales	f__Ruminococcaceae	g__Ruminococcus	s__Ruminococcus_champanellensis
46	p__Firmicutes	c__Clostridia	o__Oscillospirales	f__Oscillospiraceae	g__NK4A214_group	s__unidentified
47	p__Firmicutes	c__Clostridia	o__Lachnospirales	f__Lachnospiraceae	g__Lachnospiraceae_FCS020_group	s__mouse_gut
48	p__Firmicutes	c__Clostridia	o__Oscillospirales	f__Ruminococcaceae	g__Negativibacillus	s__unidentified
53	p__Firmicutes	c__Clostridia	o__Oscillospirales	f__Butyricicoccaceae	g__Butyricicoccus	__
55	p__Firmicutes	c__Bacilli	o__Erysipelotrichales	f__Erysipelotrichaceae	g__uncultured	s__unidentified
56	p__Firmicutes	c__Clostridia	o__Lachnospirales	f__Lachnospiraceae	g__Lachnospiraceae_UCG-006	s__Clostridium_sp.
57	p__Bacteroidota	c__Bacteroidia	o__Bacteroidales	f__Muribaculaceae	g__Muribaculaceae	__
58	p__Firmicutes	c__Clostridia	o__Oscillospirales	f__Ruminococcaceae	g__Incertae_Sedis	__
60	p__Firmicutes	c__Clostridia	o__Oscillospirales	f__Oscillospiraceae	g__UCG-005	__
62	p__Firmicutes	c__Clostridia	o__Lachnospirales	f__Lachnospiraceae	g__Lachnospiraceae_FCS020_group	s__uncultured_prokaryote
63	p__Firmicutes	c__Clostridia	o__Oscillospirales	f__Oscillospiraceae	g__uncultured	s__Clostridiales_bacterium
64	p__Firmicutes	c__Clostridia	o__Peptostreptococcales-Tissierellales	__	__	__
65	p__Firmicutes	c__Clostridia	o__Clostridia_UCG-014	f__Clostridia_UCG-014	g__Clostridia_UCG-014	s__uncultured_Acetivibrio
67	p__Firmicutes	c__Clostridia	o__Peptostreptococcales-Tissierellales	f__Anaerovoracaceae	g__Family_XIII_AD3011_group	s__gut_metagenome
68	p__Firmicutes	c__Clostridia	o__Oscillospirales	f__UCG-010	g__UCG-010	s__unidentified
69	p__Firmicutes	c__Clostridia	o__Lachnospirales	f__Lachnospiraceae	g__Lachnospiraceae_UCG-001	s__uncultured_Clostridiales
70	p__Actinobacteriota	c__Coriobacteriia	o__Coriobacteriales	f__Eggerthellaceae	g__Gordonibacter	s__uncultured_bacterium
72	p__Firmicutes	c__Clostridia	o__Oscillospirales	f__Oscillospiraceae	g__Oscillibacter	s__Clostridiales_bacterium
73	p__Firmicutes	c__Clostridia	o__Peptostreptococcales-Tissierellales	f__Anaerovoracaceae	g__[Eubacterium]_brachy_group	s__uncultured_organism
74	p__Firmicutes	c__Clostridia	o__Lachnospirales	f__Lachnospiraceae	g__A2	s__unidentified
77	p__Firmicutes	c__Bacilli	o__Lactobacillales	f__Streptococcaceae	g__Streptococcus	s__Streptococcus_danieliae
80	p__Firmicutes	c__Bacilli	o__Erysipelotrichales	f__Erysipelotrichaceae	g__Faecalibaculum	__
81	p__Firmicutes	c__Clostridia	o__Lachnospirales	f__Lachnospiraceae	g__Lachnospiraceae_NK4A136_group	s__Trichinella_pseudospiralis
86	p__Firmicutes	c__Clostridia	o__Oscillospirales	f__Ruminococcaceae	g__Candidatus_Soleaferrea	s__Ruminococcaceae_bacterium
88	p__Firmicutes	c__Clostridia	o__Oscillospirales	f__Butyricicoccaceae	g__UCG-009	s__[Clostridium]_leptum
89	p__Firmicutes	c__Clostridia	o__Lachnospirales	f__Lachnospiraceae	g__[Eubacterium]_xylanophilum_group	__
90	p__Firmicutes	c__Bacilli	o__Erysipelotrichales	f__Erysipelotrichaceae	g__Erysipelotrichaceae	s__uncultured_bacterium
105	p__Firmicutes	c__Bacilli	o__Erysipelotrichales	f__Erysipelatoclostridiaceae	__	__
